# Heavy metals accumulation in soil and uptake by barley (*Hordeum vulgare*) irrigated with contaminated water

**DOI:** 10.1038/s41598-022-18014-0

**Published:** 2023-03-13

**Authors:** Marwan Haddad, Doa Nassar, Munqez Shtaya

**Affiliations:** 1 Water and Environmental Studies Institute, An-Najah National University, Nablus, Palestine; 2 Faculty of Graduate Studies, An-Najah National University, Nablus, Palestine; 3 Department of Plant Production and Protection, Faculty of Agriculture and Veterinary Medicine, An-Najah National University, Nablus, Palestine

**Keywords:** Plant sciences, Environmental sciences, Natural hazards, Health care

## Abstract

Impacts of nine heavy metals (Cd, Cr, Cu, Ni, Pb, K, Fe, Mn, and Zn) contamination in irrigation water on the soil, shoots, and roots of barely were investigated. Due to freshwater shortages, the use of available and inexpensive urban wastewater with input from local industrial factories containing heavy metals in irrigation is still practiced in the Middle East including Palestine. Barely was grown in plastic pots filled with sandy soil irrigated with simulated treated wastewater during two growing seasons. The metal treatments investigated include one, three, nine, and 15 multiples of the average metal content of treated effluent. Results showed that (i) Barley showed similar growth responses but different metal uptake patterns, (ii) Cd, Fe, Pb, and Zn in roots and shoots of barley were higher than WHO permissible levels, (iii) all metals accumulated in the soil had lower content than WHO permissible levels, (iv) The average value of enrichment factor (EF) for most heavy metals used was around unity indicating poor enrichment to soil and translocation to roots and shoots, (v) The highest Translocation factor (TF = 57.8) and Bioconcentration Factor (BCF = 126.8) was observed for K indicating its role in enhancing barley's tolerance to drought and its effectiveness in using barley in phytoremediation, and (vi) Barley growth and development and soil quality parameters were significantly affected by repetitive and increased irrigation with wastewater containing heavy metals.

## Introduction

The importance of barley as an integral part of the farming system in Palestine (West Bank and Gaza Strip) was apparent. About 857 ha of agricultural land has been utilized for the cultivation of barley (*Hordeum vulgare* L.) in the West Bank in the year 2013/2014^1^. Barley yield in Palestine fluctuated substantially in recent years, it tended to increase through the 2000–2019 period ending at a total of 20,384 tons in 2019^2^.

The majority of barley farmers in Palestine are small-scale family farmers. They use barley as a feed for their own livestock and only 16% of them produce it as a cash crop for sale. About 12% of barley growers sold part of the total production or part of the straw^3^. Most of the cultivated barley is landraces, which have evolved directly from the wild progenitor^4^. Landraces, which have been developed over many generations by farmers’ selection for desirable traits, tend to be genetically heterogeneous and well adapted to their specific agro-ecological environment^5^.

Population growth, urbanization, and the consequence of increased industrial and agricultural production have resulted in increased generation of wastewater that contains various toxic compounds including heavy metals. Improper management of these wastewater flows have resulted in various pollution to soil and water resources in addition to health problems and risks to humans and animals.

It was indicated that irrigation water containing heavy metals and soil salinity are important environmental factors that have a serious effect on the yield, growth, and uptake of salts by barley^6–8^. Irrigation with wastewater (treated or raw) may accumulate heavy metals in the soil as well as various parts of barley including the grains, shoots, and roots. This accumulation may impact the development and growth of barley and represent a health hazard to consumers.

The accumulation of toxic heavy metals in barley depends on several factors including barley species, type of heavy metals, its bioavailability, redox, pH, cation exchange capacity, dissolved oxygen, temperature, and secretion of roots^6^.

Much research was found in the literature on evaluating heavy metals impacts and irrigation with wastewater on vegetables, trees, and soils, while inadequate publications were found on barley.

Water is an important element in all human activities including agriculture. Freshwater recourses in the world are very limited and only 0.6% of the total world water resources is freshwater^9^. Freshwater resources have been decreased at an alarming rate due to increased population growth and demand for fresh water and they may not able to meet the requirements of the different human needs in the future^10^.

The increased use of freshwater demand has resulted in an increased volume of wastewater generated. Treated wastewater is increasingly viewed as a valuable supplementary water resource for the agricultural, industrial and municipal sectors, rather than as a waste that requires disposal^11^.

In Palestine, wastewater collection systems are available in most cities. Few secondary-level wastewater treatment plants have been constructed in the past 20 years for a number of cities while raw wastewater still flowing in valleys. In many other cities such as Nablus east raw wastewater still flowing to the Jordan Valley^12^.

Many small industries are located within municipal boundaries and drain their wastewater into the municipal systems. These wastewaters contain among other pollutants appreciable amounts of heavy metals^13,14^. Several studies were conducted on the toxicity of urban wastewater in Palestine and its impacts on groundwater, plants, and soils^15–18^.

This use of wastewater in agriculture demands unique management, which in addition to its appropriate utilization, has to have no threat to the environment, plants, soils and surface, and subsurface water resources^19^.

In developing countries including Palestine, due to scarcity of fresh water and its unavailability for irrigation, the use of treated or untreated wastewater in irrigating urban horticulture is widespread^13^. This use enriched soils with heavy metals to concentrations that may pose long-term potential environmental and health risks^20^. Heavy metals are human carcinogens^21^, non-biodegradable, non-thermo-degradable, and generally difficult to leach from the topsoil^20^. It was concluded that repetitive irrigation with irrigation water containing heavy metals can induce, in long term, soil contamination which can limit plant production and enhance crop contamination^22^.

Heavy metal concentrations of urban wastewater in Palestine range from 0 to 2075 ppm for zinc, 0 to10 ppm for copper, and from 0 to 15 ppm for lead^23,24^. Zinc has the highest concentration due to the fact that zinc dissolves from galvanized steel tanks mounted on the roofs of buildings and houses as water supply storage.

Although Cu, Zn, Mn, Fe, and B are essential micro-nutrients required for plant growth, they may be toxic to plants if applied in irrigation water at high concentrations^25^. The concentrations of heavy metals in sewage effluents are usually low, but long-term application often results in the buildup of elevated concentrations of metals in soils^26^. In addition to its impacts on the plants themselves, the application of high heavy metal concentrations might pollute groundwater and soil^27^.

18it was found that phytoremediation, the use of plants to clean up toxic heavy metals, can be used to remove heavy metals from contaminated soils and sites^28,29^.

The objective of the present study was (1) to assess the effects of long-term irrigation with sewage effluents on metal contents in soil and barley plants, and (2) to determine Enrichment (EF), Bioconcentration (BCF), and Translocation (TF) Factors and their importance to barley and its metal uptake.

## Materials and methods

### Experimental site and program

The experiment was conducted under controlled conditions in the greenhouse at the Faculty of Agriculture and Veterinary Medicine, An-Najah National University, Tulkarm (Khadouri) campus, during one growing season (2013/2014). The experiment was carried out under greenhouse conditions to avoid any variation in the experimental site. All experiments and lab analyses were conducted in compliance with pertinent Standards promulgated by Palestinian Standard Institution (PSI including PS-3081, PS-3138, PS-3139, and PS-3158.

**Figure 1:**
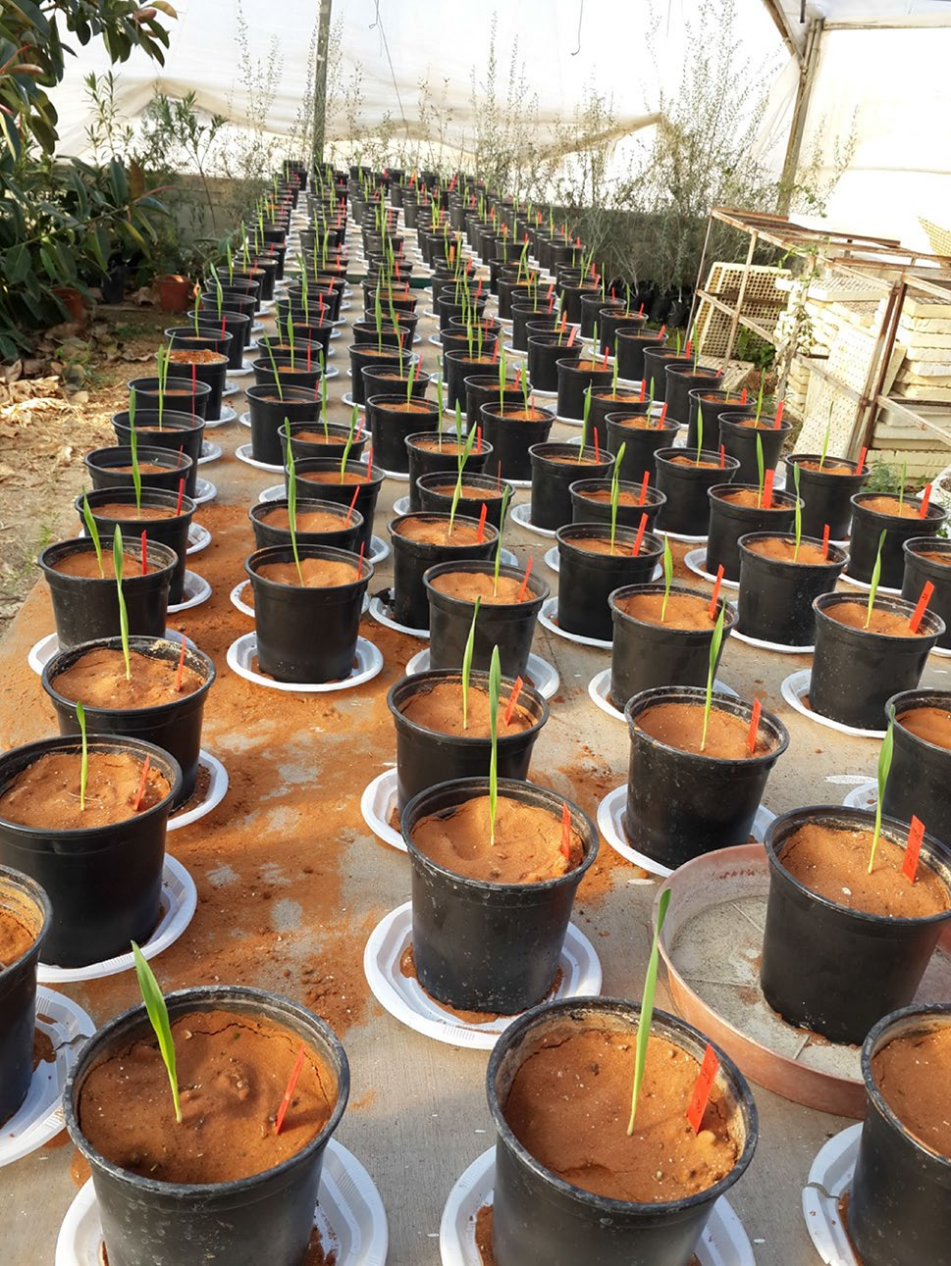
Picture of barley plants at one leave germination.

Four metal treatments were used to simulate one, three, nine, and 15 years of irrigation application. An additional blank (B) pots planted with the same barely landraces and irrigated with tap water containing no heavy metals were used as a reference sample (control). The experiment was arranged in a randomized complete block design (CRD) with four replicates to reduce the residual variance and the average standard error of a difference (see Table 1 below). The number of pots per block per treatment was 15 pots (one plant per pot). With four treatments plus blank, four replicates, and nine heavy metals, this makes the total number of pots used was 180 pots. Figure 1 below shows a picture of the pots at one leave germination set in the greenhouse.

**Table 1:** Randomized complete block design arrangement of barley pot experiment.

9X	X	3X	15X	B
3X	15X	B	X	9X
15X	B	X	9X	3X
X	3X	9X	B	15X

The concentration level of heavy metals in irrigation wastewater in this experiment, (X), was simulated to equal the average quality of the effluent from the full-scale wastewater treatment facilities of the Nablus West treatment plant. Lead (Pb), Cadmium (Cd), Chrome (Cr), Zinc (Zn), Iron (Fe), Copper (Cu), Manganese (Mn), Nickel (Ni), and potassium (K) added to tap water in different concentration (X) and 3, 9, and 15 multiples as showed in Table 2.

**Table 2:** Simulated heavy metal concentration in irrigation water [ppm].

Element	X	3X	9X	15X	Composition
K	15.0	45.0	135	225.0	KCl
Zn	0.1	0.3	0.9	1.5	Zn metal
Cu	0.2	0.6	1.8	3.0	CuSO_4_
Fe	0.1	0.3	0.9	1.5	FeCl_2_
Mn	0.02	0.06	0.18	0.3	KMnO_4_
Ni	0.02	0.06	0.18	0.3	NiCl_2_
Pb	0.02	0.06	0.18	0.3	Pb metal
Cd	0.02	0.06	0.18	0.3	Cd metal
Cr	0.02	0.06	0.18	0.3	Cr metal

### Plant material and growth

The experiment was carried out using the main local barley landrace cultivated by the farmers in the West Bank, Palestine. A single seed descent population was produced and used across the experiment. Barely was sown on the 1st of November in two-liter size plastic pots filled with agricultural sand. Agricultural sand was used in order not to stick with plant roots. As a result, the heavy metals that will be absorbed from the irrigated water will be conservative. In this experiment, the planted seeds were irrigated two times a week, 50 ml each until germination. After that, the irrigation requirements were calculated using the average climatic parameters of the Tulkarm area. During the growing season, the number of leaves and plant height were recorded for each landrace at a two weeks interval. At maturity, samples were collected from each accession including shoot, roots, and soil. Collected samples were stored separately in a paper bag for chemical analysis.

### Heavy metal content

Heavy metal content in plant tissues and soil was performed on collected samples in the laboratory using Inductively Coupled Plasma Mass Spectrometry (ICP-MS) according to procedures and methods listed in: analysis of major, minor and trace elements in plant tissue samples with ICP-OES and ICP-MS^30,31^**.**

### Statistical analysis

Two-way analysis of variance (ANOVA) for RCBD was performed on field data (number of leaves and height of plant) and on laboratory data (concentrations of heavy metals) using GLM procedure of SAS STAT software, means were obtained and multiple comparisons among pairs were performed using the Duncan-test (*p* ≤ 0.05).

## Results and discussion

The results were presented in five sections including Plant development and growth, Trace Metals Concentration in Plant (Vegetative Part), Trace Metals Concentration in Roots, Trace Metals Concentration in Soil, and Impact Evaluation Factors. An additional general discussion was added at the end. The discussion of the results of the 15X treatment was omitted due to the large differences and the greater effect of the high concentration used.

### Plant development and growth

**Figure 2:**
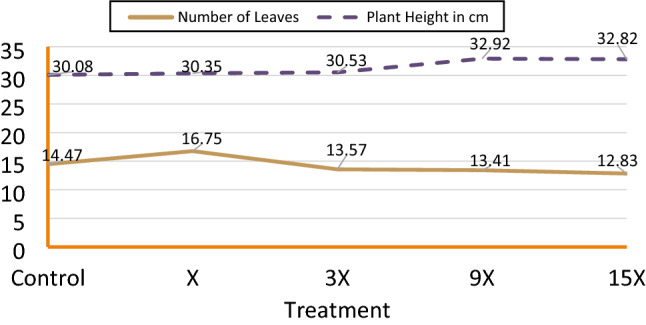
Plant growth responses to different metal applications.

The average number of leaves and plant height were recorded and presented in Fig. 2. It was observed that barley plants with different metal applications showed similar growth responses with little variations. However, as will be shown later that different metal uptake patterns were observed for the four different treatments. In a parallel study, the authors found that growing strength as well as the growth period (from days to emergence to maturity) were not affected by the type of irrigation water and only depend on the genotype^17^.

### Trace metals concentration in plant (vegetative part)

Average values for element concentrations (mg.kg^−1^ dry weight or ppm) in the barley plant vegetative parts are presented in Table 3. Results showed the following:

- Cadmium (Cd), Chromium (Cr), and Lead (Pb) concentration in vegetative parts of barley had an increasing trend with increased metal application in irrigation water. These three metals have no nutritious capacity, but they are exceptionally toxic.
- The response of other metals in barley showed no systematic relationship to metal application. González and Lobo, (2013) reported similar results for barley planted in soils contaminated with heavy metals^32^.
- Cadmium, Chrome, and Lead accumulation concentrations in barley were greater than the permissible level set by WHO for these metals^33^, and accordingly these plants are not suited for consumption. Another metal accumulation concentration was within permissible levels set by WHO. However, this high metal accumulation level did not affect plant growth appearance, and/or yield, a result agreed by Bigdeli and Seilsepour^34^.
- Ferrous (Fe), and Manganese (Mn) concentrations were higher in the control compared to all metal-treated plants. This indicates that Fe and Mn accumulation in barley is not related to its metal application rate.
- Results obtained in this study agree with published research which indicated that for Cu, Zn, Fe, Mn content accumulated in barley plants irrigated with wastewater containing heavy metals was high up to 2 years of irrigation with heavy metals, and the content of these metals was reduced for longer irrigation than 2 years (5–10 years)^35^. Similar higher accumulation of

- Fe (129–968 ppm) and Mn (19–137 ppm)^36^
- Fe (116–378 ppm) and Mn (12–69 ppm)^37^
  was found in vegetables irrigated with wastewater.

- No specific trend or relationship was found for the repetitive and multiple applications of Cu, K, Ni, Pb, and Zn and their accumulation in barley.
- The decreasing order of the maximum average concentration of heavy metals in barley vegetative parts was: K (342,926 ppm) > Fe (881.88 ppm) > Mn (397.22 ppm) > Zn (131.75).

**Table 3:** Average heavy metals concentrations in plant (vegetative part) [ppm].

Treatment	Cd	Cr	Cu	Fe	K	Mn	Ni	Pb	Zn
Control	0.54^b^	4.13^c^	36.18b^c^	1003.78^a^	354070^a^	469.96^a^	5.31^b^	3.66^b^	129.07^a^
X	0.74^b^	5.31^c^	48.40^a^	943.04^ab^	354070^a^	442.00^ab^	9.88^ab^	4.50^b^	124.25^a^
3X	0.83^ab^	6.59^c^	26.48^c^	750.33^b^	354070^a^	322.79^b^	12.30^a^	3.77^b^	159.61^a^
9X	0.95^ab^	15.49^b^	43.37^ab^	751.79^b^	354070^a^	427.47^ab^	6.29^b^	4.85^b^	139.55^a^

Means in the same column with similar superscripts are not statistically different (Duncan test, *P* ≤ 0.05).

### Trace metals concentration in roots

The average values of heavy (trace) elements in plant roots are presented in Table 4. In general, there was an increase in metal concentration accumulation in barley's roots as a response to the increased metal application in irrigation water. For Cd, Cu, Mn, Ni, Pb, and Zn metal accumulation in the roots of the control was greater 'than that in some metal treatments. This result agrees with published data for different plants^32,34,38,39^.

**Table 4:** Average heavy metals concentrations in root [ppm].

Treatment	Cd	Cr	Cu	Fe	K	Mn	Ni	Pb	Zn
Control	0.11^b^	10.80^ cd^	10.28^c^	2566.60^b^	4530^b^	156.30^b^	13.33^ab^	4.44^bc^	45.87^b^
X	0.03^b^	5.88^d^	7.83^c^	1434.80^c^	4852^b^	59.68^c^	6.56^c^	1.94^d^	20.50^c^
3X	0.12^b^	14.85^c^	10.10^c^	1569.30^c^	6297^ab^	92.90^c^	8.09^c^	3.34^ cd^	77.48^a^
9X	0.73^a^	68.94^a^	19.60^a^	3384.40^a^	8022 ^a^	211.22^a^	15.13^a^	5.73^b^	50.89^b^

Means in the same column with similar superscripts are not statistically different (Duncan test, *P* ≤ 0.05).

In general, the accumulation concentration of heavy metals in roots increased consistently with the metal total contents in the soil.

Although accumulated Cu levels increased in both root and shoot in response to the metal application in irrigation water, Cu accumulation concentration in shoots increased more sharply than that in roots (see Table 4). It was reported^40^ that Cu concentration in the shoots was significantly influenced by Cu concentration in soil and increased markedly with an increase in the soil Cu concentration. González and Lobo^32^ found that Zn concentration was higher in the roots of four varieties of barley than in other parts of the plant.

The metal accumulation concentration of Cr and Fe was always higher in roots than in the vegetative parts. A similar result for the accumulation of Cr was reported^41^. This could be because Cr is immobilized in the vacuoles of the root cells, which may be a natural toxicity response of the plant^41^. For Fe, Chiroma et al. (2014) reported similar results in Bush green and Roselle plants^42^.

### Trace metals concentration in soil

The average values of heavy (trace) elements in the soil are presented in Table 5 Metal accumulation in the control treatment showed higher accumulation concentration for all elements except for Cd and Zn.

**Table 5:** Average heavy metals accumulation concentrations in soil [ppm].

Treatment	Cd	Cr	Cu	Fe	K	Mn	Ni	Pb	Zn
Control	0.07^b^	50.47^a^	19.19^a^	30812^a^	6775^a^	508.37^a^	35.36^a^	20.17^a^	94.10^a^
X	0.03^b^	9.21^c^	5.73^c^	6204^b^	2239^b^	106.20^bc^	8.29^c^	5.22^c^	35.90^a^
3X	0.00^b^	13.77^c^	5.62^c^	7393^b^	1893^b^	135.68^bc^	9.04^c^	4.97^c^	35.90^a^
9X	0.33^a^	32.96^b^	12.09^b^	15408^b^	6191^a^	269.17^b^	20.90^b^	12.69^b^	1607.80^a^

Means in the same column with similar superscripts are not statistically different (Duncan test, *P* ≤ 0.05).

In general, no systematic accumulation in soil was observed for all metal treatments. Also, accumulation concentration in the soil beyond irrigation with 9X treatment was reduced for all metal applications which indicates a limiting accumulation capacity of the soil.

No significant differences were observed for Ferrous (Fe) and Zinc (Zn) accumulation between X, 3X, and 9X. This agrees with the results reported by several researchers^35,43,44^.

The highest concentration was found in soils irrigated with tap water except for Cd and Zn. This indicated that originally the soil contains elevated amounts of these elements and irrigation leached these elements down the soil profile. Previous studies reported that the movement of heavy metals in soils irrigated with wastewater is very slow^43,45^. Other researchers showed that the amount of metal that remained in the soil was greatest for the higher concentration of Zn and Cd treatments^32^.

The iron was found to be higher in comparison to all other studied metals in soil. This result agrees with the findings of Zafar et al.^46^.

All heavy metals had concentrations in soil marginally below the WHO maximum permissible level meaning that there is no threat of soil contamination by these metals when the effluent of the treatment plant is used for the irrigation of barely. A result indicating the appropriateness of sandy soils to minimize health and environmental risk hazards associated with irrigation with water containing heavy metals. This agrees with results reported by other researchers^34,42,43^.

The highest Cd, Cu, and K concentration for these metals was in the vegetative parts of barley while the lowest was in soil.

It is very important to note that in reality soils used to plant barley are mostly not sandy as used in this study and therefore geochemical monitoring of and evaluation of heavy metals in other soils is important and necessary to ensure proper barley agricultural production and quality.

The average heavy metal concentrations in soils were observed in the following decreasing order: Fe > K > Zn > Mn > Cr > Ni > Pb > Cu > Cd. This sequence follows to some extent a natural progressive concentration of heavy metals in sandstones^47,48^.

### Impact evaluation factors

To understand soil contamination and the ability of barley or part of it to uptake/accumulate metals and to assess the plant’s potential for phytoremediation purposes in response to metal inputs from anthropogenic sources several factors were investigated including enrichment factor (EF), bioconcentration factor BCF), and translocation factor (TF). The three factors were estimated based on dry weight concentration in ppm.

#### Enrichment factor

Enrichment factor (EF) was used/estimated in order to derive the degree of soil contamination and heavy metal accumulations in soil and in plants irrigated with heavy metal contaminated water. EF was defined as the ratio of metal concentration in the plant of contaminated soil to plant of uncontaminated soil (control), were listed in Tables 6 and 7 and showed the following development:

- For Cd, Cr, Cu, Fe, K, and Ni there was an increasing trend of the EF in roots of barley observed with treatment until the 9X treatment then with higher metal treatment the EF was reduced.
- In general, small variations of the EF in shoots of barley for all metals were observed.
- The EF in shoots and roots indicating the no effect or relationship of increasing treatment level beyond 9X on all metal accumulation.
- As most of the EF for all metals were less than 2, an indication that (i) the soil used in the experiment was deficient in minimal enrichment (which is normal for sandy soils) and (ii) the soil was moderately contaminated upon various levels of metal applications (treatment). This result agrees with the findings of^47,48^.
- The overall average of EF for shoots (1.13) was less than that of roots (1.47) indicating higher enrichment of metals in barley's roots than in shoots.

**Table 6:** Enrichment factors (EF) of shoots of barley.

Treatment	Cd	Cr	Cu	Fe	K	Mn	Ni	Pb	Zn
X	1.38	1.28	1.34	0.94	1.00	0.94	1.86	1.23	0.96
3X	1.13	1.24	0.55	0.80	1.00	0.73	1.25	0.84	1.28
9X	1.14	2.35	1.64	1.00	1.00	1.32	0.51	1.29	0.87
Average	1.25	1.52	1.11	1.10	0.96	0.94	1.16	1.25	0.97

**Table 7:** Enrichment factor in barely roots.

Treatment	Cd	Cr	Cu	Fe	K	Mn	Ni	Pb	Zn
X	0.27	0.54	0.75	0.57	1.01	0.38	0.50	0.43	0.43
3X	3.77	2.52	1.29	1.09	1.30	1.56	1.23	1.73	3.78
9X	5.97	4.64	1.94	2.16	1.27	2.27	1.87	1.71	0.66
Average	2.27	2.04	1.17	1.17	1.12	1.24	1.09	1.31	1.41

The average value of enrichment factor (EF) for Cd (2.27) and Cr (2.04) indicated moderate to weak enrichment in barley irrigated roots. The EF of the rest of the heavy metals used were around unity indicating poor enrichment to soil and translocation to roots and shoots.

#### Bioconcentration factor

To better evaluate the ability of barley to accumulate within its tissues the heavy metals from simulated treated wastewater, this study used a bioconcentration factor (BCF) to measure the absorption capacity of the heavy metals by barely. The values of BCF defined as the ratio of metal content in plants to the total content in soil are shown in Tables 8 and 9. In general, if the BCF is ≤ 1, then the value denotes that barley is able to absorb the heavy metals, but does not accumulate them within its tissues, accumulation within tissues occurs when BCF > 1. According to the FAO/World Health Organization (WHO)^49,50^, grains and cereals with BCF higher than 0.20 are thought to be highly contaminated by anthropogenic activities and have a high health risk.

**Table 8:** Bioconcentration factor (BCF) in shoots.

Treatment	Cd	Cr	Cu	Fe	K	Mn	Ni	Pb	Zn
Control	5.99	0.07	1.68	0.03	40.65	0.74	0.14	0.17	1.13
X	21.38	0.58	8.45	0.15	158.16	4.16	1.19	0.86	3.84
3X	0.00	0.48	4.71	0.10	187.05	2.38	1.36	0.76	4.45
9X	2.87	0.47	3.59	0.05	57.20	1.59	0.30	0.38	0.09
Average	6.05	0.80	6.43	0.13	126.8	2.99	0.88	0.94	2.06

**Table 9:** Bioconcentration factor (BCF) in roots.

Treatment	Cd	Cr	Cu	Fe	K	Mn	Ni	Pb	Zn
Control	1.35	0.19	0.48	0.06	0.55	0.25	0.34	0.20	0.42
X	0.93	0.64	1.37	0.23	2.17	0.56	0.79	0.37	0.63
3X	0.00	1.08	1.80	0.21	3.33	0.68	0.89	0.67	2.16
9X	2.19	2.09	1.62	0.22	1.30	0.78	0.72	0.45	0.03
Average	0.89	1.59	2.00	0.33	2.38	1.03	1.05	0.85	0.71

The order of the bioconcentration factor (BCF) measured in barley's shoots was K > Cu > Cd > Mn > Zn > 1, indicating that barley possessed a strong biological enrichment ability to accumulate a variety of heavy metals.

Potassium in shoots showed the highest BCF with an increasing trend to metal application followed by Cd and Cu while the rest of the metals showed low BCF values. BF in roots was low for all metals. BF data in shoots and roots suggested that no direct relationship exists between BF and metal concentration in irrigation water. BCF values greater than 1 were observed in shoots and roots for most metals and applications indicating the ability and potential success of barley for phytoremediation.

BCF values listed in s for shoots and roots indicated that Cd, Cr, Cu, K, Mn, Ni, and Pb transfer from soils to plants is directly correlated with the available metal concentration in soils.

It was observed that BCF is a variable for the various metals and applications used in this study. BCF in shoots and roots was most directly related to metal application concentrations in irrigation water.

The Cr, Fe, and Pb little build-up in the shoots and roots of barley with BCF << 1 could be related to soil type but are issues that need further verification in future studies.

The overall average of BCF for shoots (19.02) was much greater than that of roots (1.40) indicating higher metal transferability, accumulation, and buildup took place in the barley's shoots despite the high application of metals to the soil. However, the low BCF root values and averages were impacted by the sandy soil used in the experiment as a planting medium.

BCF in shoots was the highest in K (53.79), followed by Cd (8.24), and then Mn, Cu, and Zn (3.75, 3.48, 3.38), while the lowest was Fe (0.42), according to the overall averages.

#### Translocation factor

The Translocation factor (TF) is defined as the ratio between the metal content in shoots and that in roots which explains the ability of barley plant to translocate metals from roots through shoots and leaves which is primarily responsible for phytoextraction TF values obtained were listed in Table 10 and showed the following development:

- The TF for K was the highest among all metals (>> 1) and showed a decreasing trend with increasing treatment. It was found that Potassium plays a major role in enhancing the tolerance of barley to drought by increasing translocation and maintaining water balance^51^. High TF values with less extent were also observed for Cd, Cu Mn, and Zn. For Cd, Cr, Cu, Fe, K, and Ni there was an increasing trend of the EF in roots of barley observed with treatment until the 9X treatment then with higher treatment the EF was limited.
- In general, no specific trend of the TF was observed for other metals and treatments.
- TF values of >> 1 in Cd Cu, K, Mn, and Zn indicate that barley is considered a strong accumulator of the corresponding metals. It also indicates that sandy soil is not suitable for barley stabilizing these metals in soil.

**Table 10:** Translocation factor.

Treatment	Cd	Cr	Cu	Fe	K	Mn	Ni	Pb	Zn
Control	4.45	0.38	3.48	0.40	73.87	3.00	0.40	0.82	2.70
X	22.92	0.90	6.18	0.66	72.97	7.41	1.51	2.32	6.06
3X	6.84	0.44	2.62	0.48	56.23	3.47	1.52	1.13	2.06
9X	1.31	0.22	2.21	0.22	44.14	2.02	0.42	0.85	2.74
Average	7.48	0.51	3.48	0.42	57.8	3.60	0.88	1.23	3.25

According to Mellem et al.^52^, transfer factor values nearer to zero imply high retention of metal in the soil and result in less movement to the plants. Thus, the low level of TF observed in the case of Cr (TF 0.22–0.90), and Fe (TF 0.22–0.66), implies that only a small portion will be available in the plant’s vegetative parts. This result agrees with the enrichment factors in shoots and roots for these metals in Tables 6 and 7. However, TF values for all other heavy metals were much greater than unity, indicating high mobility of these metals from soil and roots to the vegetative parts of barley and thus labeling barley as a heavy metal accumulator. We can consider barley as a potential candidate for phytoextraction and phytoremediation of soils contaminated with these metals.

### General discussion

To show metal movement and differentiation between soil, roots, and vegetative parts of barley (shoots and leaves), the values overall heavy metals averages for all treatments are listed in Table 11.

**Table 11:** Overall heavy metals averages for all treatments, [ppm].

Treatment	Cd	Cr	Cu	Fe	K	Mn	Ni	Pb	Zn
Vegetative Parts	0.87	10.01	38.82	882	342,926	397.2	8.01	4.94	131.8
Roots	0.33	26.3	12.3	2571	6168	135.0	10.9	4.67	46.9
Soil	0.086	22.8	9.1	12,574	3732	214.6	15.6	9.2	381.4
FAO/WHO*	0.20	2.30	73.3	425.5	300	0.20	67.9	0.30	99.4

Vegetative Parts = Shoots + Leaves.

* = in vegetables.

As shown in Table 11, heavy metals (Cd, Cr, K Mn, and Pb) were mainly concentrated in the vegetative parts of barley while Cu, Fe, Ni, and Zn were concentrated in the roots of barley indicating its different extraction, biological enrichment, and accumulation ability to accumulate a variety of heavy metals. Also, the extent of heavy metals accumulation was different by the different heavy metals.

A systematic increasing trend of heavy metals, Cd, Cu, K, and Mn accumulation from soil (lowest) to shoots and leaves (highest). Other metals did not show such a trend. The overall average concentration of most heavy metals in barley's vegetative parts (Table 11) exceeds the maximum FAO/WHO permissible values. These metals showed strong enrichment capacity in barley shoots (Table 6). For such heavy metals, barley proved to be suitable to be used as a phytoremediation method for wastewater treatment.

The overall average of Cd soil, roots, and vegetative parts was 0.08, 0.33, and 0.87 ppm, respectively showed an increasing trend and most importantly large differences between the three parts: four times in roots than soil and eleven times in the vegetative parts than soil. This proves the ability of the barley plant to translocate Cd from the soil through roots to shoots and leaves.

Only Cd and Ni overall average concentrations in soil were below the WHO/FAO maximum permissible level of 0.2 and 67.9 ppm, respectively.

The overall average of Cr for the five treatments in soil, roots, and shoots was 22.8, 26.3, and 10.0 ppm, respectively indicating no systematic changing trend and a threefold difference between the three parts. All concentrations were larger than the WHO/FAO permissible level of 2.3 ppm for Cr.

The overall average of Cu for the five treatments in soil, roots, and shoots was 9.1, 26.3, and 38.8 ppm, respectively indicating an increasing trend and large difference between the three parts. Only All concentrations were below the WHO/FAO permissible level of 73 ppm for Cu.

Among various metallic concentrations, Fe had the highest concentration. Fe maximum concentration was observed in soils (12,574 ppm), then in the root (2571 ppm), and the least in the vegetative parts of barley (882 ppm). The bioaccumulation factor values for Fe in shoots and roots were << 1 indicating the inability of barely to be suitable for removing Fe.

The average EF for soil for all metals in shoots was 1.52–0.94 indicating deficient or minimal enrichment of the soil. differed depending on the type and concentration of the effluents and the type of soil. Similar results were obtained for the average EF for soil for all metals in roots were 2.27–1.12.

According to Cluis^53^, hyperaccumulating plants have a BCF value of more than 1.0. In this study,

$${\text{BCF in shoots }} > { 1}:{\text{ Cd 6}}.0{5},{\text{ Cu 6}}.{43},{\text{ K 126}}.{8},{\text{ Mn 2}}.{99},{\text{ and Zn 2}}.0{6}$$

$${\text{BCF in roots }} > { 1}:{\text{ K 2}}.{38},{\text{ Cu 2}}.0,{\text{ Cr 1}}.{59},{\text{ Mn 1}}.0{3},{\text{ and Zn 1}}.0{5}$$

Only Cd, Cu, and K in shoots have relatively high BCF and are hyperaccumulating. The lower BCF values in roots might be related to soil metal composition.

The average translocation factor (TF) for K > Cd > Mn > Cu > Zn > Pb > 1 indicates the ability of barley to translocate these heavy metals to shoots and leaves.

The translocation factor (TF) values decreased in the order of Ni > Cr > Fe, all of which were lower than 1, which showed that the absorption of these heavy metals by barley was mainly accomplished in the roots.

It is important to note that it is difficult to assess the impact of each heavy metal separately due to the joint application of metals. A deficiency of this research and a recommendation for future works.

The overall risk calculation of the three factors reveals that

- Hyperaccumulation of metals in shoots, Cd, Cu, and K
- High metal translocation from soil to shoots indicates the suitability of barley to be used as a phytoremediation agent or plant
- Barely irrigated with treated effluent containing heavy metals is unsafe for human or animal consumption
- On the long-term metal accumulation in barley increases, increasing plant toxicity
- The is no flexibility or possibility of no metal accumulation in barley upon its irrigation with wastewater containing heavy metals

And therefore, the health and environmental risk are high for barley's consumers and water and land resources.

To evaluate the long-term human health risk of exposure of cows and sheep to carcinogen heavy metals in barley grown/irrigated with simulated treated wastewater via ingestion, the following equations were used:

$${\text{HQingest }} = \frac{{{\text{C }} \times {\text{ IRingest }} \times {\text{ EF }} \times {\text{ ED}}}}{{{\text{BW }} \times {\text{ AT }} \times {\text{ RfD}}}}$$

To assess the overall carcinogenic effects of exposure to multiple carcinogen heavy metals via different pathways, the sum of the HQ values representing the health hazard index of all heavy metals was estimated^54,55^:

$${\text{Hazard Index }}\left( {{\text{HI}}} \right) \, \sum {\text{n i}} = \sum {{\text{HQ}}}$$

$${\text{Health Risk }} = {\text{ HI }}*{\text{ SF}}$$

where AT is the averaging time, days = lifetime × 365 days; IR the daily ingestion rate, kg/day; EF is the exposure frequency (days/year); ED is the exposure duration (years); BW animal weight (kg); C is the concentration of heavy metal in vegetative parts, ppm (mg/kg); RfD is the reference oral dose, (mg/kg bw/day) ; HRI = Hazard risk index; HQ = Hazard Quotients; HI = Health Index; SF = carcinogenic slope factor (SF).

The carcinogen heavy metals in this study simulated wastewater were Cd, Pb, and Ni. An indicative estimation of hazard quotients and health index for carcinogen heavy metal exposure of cows and sheep from ingesting vegetative parts of barley was presented in Table 12. As shown in Table 12 the estimated health index for cows was 7.49 >> 1 and for sheep was 28.38 >> 1 which indicates without multiplying it with the carcinogen slope factor SF to obtain the health risk (SF for Cd = 6.1) that the vegetative parts of barley grown and irrigated with simulated wastewater in this study are carcinogen for cows and sheep and should not be used for feeding them.

**Table 12:** Estimation of hazard quotients and health index for carcinogen heavy metal exposure of cows and sheep from ingesting vegetative parts of barley.

Metal	C veg parts, ppm	RfD mg/kg-day	IR, kg/day	BW, kg	ED year	EF days/yr	AT, days	HI for Cows
Cd	0.87	0.0005	2	910	2	350	730	3.67
Ni	8.01	0.02	2	910	2	350	730	0.84
Pb	4.94	0.038	2	910	2	350	730	2.97
Total								7.49

Metal	C veg parts	RfD	IR	BW	ED	EF	AT	Hqi for Sheep
Cd	0.87	0.0005	0.5	60	2	350	730	13.9
Ni	8.01	0.02	0.5	60	2	350	730	3.2
Pb	4.94	0.038	0.5	60	2	350	730	11.28
Total								28.38

Data Source:^56–58^.

## Concluding remarks

Based on the results obtained in this study, the following concluding remarks were derived:

- Soil and crop quality parameters are significantly affected by long-term wastewater irrigation.
- Sandy soil was a marginal accumulator of heavy metals with minimum environmental risk while barley was a strong heavy metal accumulator.
- Heavy metal accumulation differed according to the part of the plant.
- Barley under investigation in this study are heavy metal accumulators and thus unsafe for consumption.
- Continuous irrigation with treated wastewater may lead to the accumulation of heavy metals beyond crop tolerance and permissible levels.
- BCF factor indicated that barley possessed a strong biological enrichment ability to accumulate a variety of trace elements.
- Cd, Fe, and Pb concentrations in plant organs (shoots and roots) were noticeably larger than the WHO standards.
- Most translocation factors were higher than 1, indicating that the barley is suitable for phytoremediation.
- Metals Cr, Fe, Ni, and Pb need further investigation on suitability for phytoremediation.
- Proper management of wastewater irrigation and periodic monitoring of soil fertility and quality parameters are required to ensure successful, safe, and long-term reuse of wastewater for irrigation.
- No relation was found between barley's growth and appearance and metal accumulation in its parts.
- Given the importance of the environmental risks of contamination of the human food chain via plant products and based on our results, the irrigation with wastewater containing heavy metals must be more controlled and restricted.
- The vegetative parts of barley grown and irrigated with simulated wastewater in this study are carcinogens for cows and sheep.

## Data Availability

All data that support the findings of this study have been included in the Result and Discussion section.
